# Effects on Oral Squamous Carcinoma Cell Lines and Their Mechanisms of Pyrazole N-Aryl Sulfonate: A Novel Class of Selective Cyclooxygenase-2 Inhibitors

**DOI:** 10.3390/ijms26188906

**Published:** 2025-09-12

**Authors:** Shiqi Wang, Mingxuan Shi, Huihui Wang, Xianlin Zeng, Dingtai Zhang, Zhiyuan Zhang, Zhaoqing Xu, Yi Li

**Affiliations:** 1 Key Laboratory of Dental Maxillofacial Reconstruction and Biological Intelligence Manufacturing, School of Stomatology, Lanzhou University, Lanzhou 730030, China; wangshq2020@lzu.edu.cn (S.W.); shimx2024@lzu.edu.cn (M.S.); wanghhlzu@163.com (H.W.); zengxl2017@163.com (X.Z.); zhangdt2024@lzu.edu.cn (D.Z.); zhzhiyuan2024@lzu.edu.cn (Z.Z.); 2 Key Laboratory of Preclinical Study for New Drugs of Gansu Province, School of Basic Medical Sciences, Lanzhou University, Lanzhou 730030, China

**Keywords:** pyrazole N-aryl sulfonate derivatives, COX-2, oral squamous cell carcinoma, non-steroidal anti-inflammatory drugs, anti-tumor therapy

## Abstract

Oral squamous cell carcinoma (OSCC) is a highly aggressive malignancy with limited effective treatment options. This study aimed to explore the therapeutic potential of novel pyrazole N-aryl sulfonate derivatives (compounds **4b**, **4d**, and **5f**) as selective cyclooxygenase-2 (COX-2; prostaglandin-endoperoxide synthase 2, PTGS2) inhibitors in OSCC. Using CCK-8 and Transwell assays, we evaluated the anti-proliferative and anti-migratory effects of these compounds on CAL-27 and SAS cell lines, while apoptosis was assessed by Hoechst 33342 staining and flow cytometry. Molecular mechanisms were investigated through RT-qPCR, Western blot, and ELISA, focusing on COX-2, MMP2, MMP9, BCL2, BAX, and the JAK/STAT3 pathway. The results demonstrated that compounds **4b**, **4d**, and **5f** significantly inhibited cell proliferation and migration, induced apoptosis, and downregulated the expression of COX-2 and its downstream targets. Notably, these compounds exhibited lower cytotoxicity in VERO cells, indicating favorable biological safety. In conclusion, our findings suggest that pyrazole N-aryl sulfonate derivatives effectively suppress OSCC cell growth and migration by targeting COX-2 and the JAK/STAT3 pathway, highlighting their promise as potential targeted therapeutics for OSCC.

## 1. Introduction

Oral squamous cell carcinoma (OSCC), one of the most prevalent malignant tumors in the head and neck region, is characterized by rapid progression, high aggressiveness, and poor 5-year survival rates [1,2,3,4]. Current standard therapeutic protocols primarily involve surgical intervention combined with radiotherapy and chemotherapy, yet these approaches are associated with significant limitations, including severe systemic toxicity, suboptimal therapeutic outcomes, and drug resistance [5,6]. Consequently, the development of safe and effective treatment modalities for OSCC, particularly highly efficacious and non-invasive pharmaceutical agents, holds critical importance for improving clinical management and treatment outcomes in OSCC patients [7,8,9].

With in-depth investigations into cancer mechanisms, COX-2 has been identified as being overexpressed in numerous cancer types, where it exerts pleiotropic and multifaceted roles in tumorigenesis, neoplastic progression, and cancer cell resistance to chemotherapy and radiotherapy [10,11,12]. COX-2 is secreted into the tumor microenvironment (TME) by cancer-associated fibroblasts (CAFs), alternatively activated macrophages (M2 macrophages), and cancer cells [13,14,15]. It sustains cancer stem cell (CSC) properties and promotes apoptosis resistance, proliferation, angiogenesis, inflammation, invasion, and metastasis of malignant cells, predominantly through the biological activities of its catalytic product prostaglandin E2 (PGE2) [16,17,18]. Extensive experimental, epidemiological, and clinical investigations into COX-2 have established the antitumor potential of non-steroidal anti-inflammatory drugs (NSAIDs), particularly highly selective COX-2 inhibitors, as a prominent research focus in recent years [19,20,21]. Among these selective inhibitors, celecoxib (CXB), a novel NSAID and potent selective COX-2 inhibitor, has demonstrated therapeutic efficacy in the prevention and management of various malignancies [22,23,24]. Mechanistically, it competitively occupies the COX-2 catalytic site, preventing substrate binding and suppressing prostaglandin biosynthesis [25,26]. Beyond this primary mechanism, its antitumor effects encompass multifaceted pathways, including inhibition of tumor cell proliferation, induction of apoptosis, suppression of telomerase activity, blockade of angiogenesis, reduction of invasive and metastatic capacities of cancer cells, and sensitization to chemo-radiotherapy [26,27]. From a dosimetric perspective, a broad body of experimental evidence indicates that the antitumor ${\mathrm{IC}}_{50}$ of CXB lies between 10 and 100 μM [28,29,30]. Furthermore, clinical studies have also revealed that COX-2 inhibitors synergize with chemotherapeutic agents to induce apoptosis in hepatocellular carcinoma cells [31,32]. Collectively, these genetic and pharmacological findings unveil the therapeutic potential of selective COX-2 inhibitors in cancer management, thereby providing novel insights for OSCC treatment strategies [33,34,35,36,37].

Building upon the therapeutic potential of COX-2 inhibitors, we designed a novel series of pyrazole N-aryl sulfonate derivatives structurally inspired by CXB. Utilizing an innovative synthetic method developed by our group, these compounds were engineered to retain potent COX-2 inhibitory activity while avoiding sulfonamide-related allergic reactions [38]. In previous experiments, preliminary screening revealed concentration-dependent antiproliferative effects of compounds **4b**, **4d**, and **5f** on HepG2, accompanied by suppression of migration and induction of apoptosis.

Based on these findings and suggestive hypotheses, compounds **4b**, **4d**, and **5f** were selected alongside the reference drug CXB for subsequent molecular biological research in OSCC cell lines, such as proliferation, migration, and apoptosis, and to further clarify the related mechanisms of cancer inhibition. The objective is to screen out selective COX-2 inhibitors that can be applied in the clinical treatment of OSCC from these compounds and provide experimental references for the subsequent development of new drugs.

## 2. Results

### 2.1. Molecular Docking of Compounds **4b**, **4d** and **5f** with COX-2

**Figure 1:**
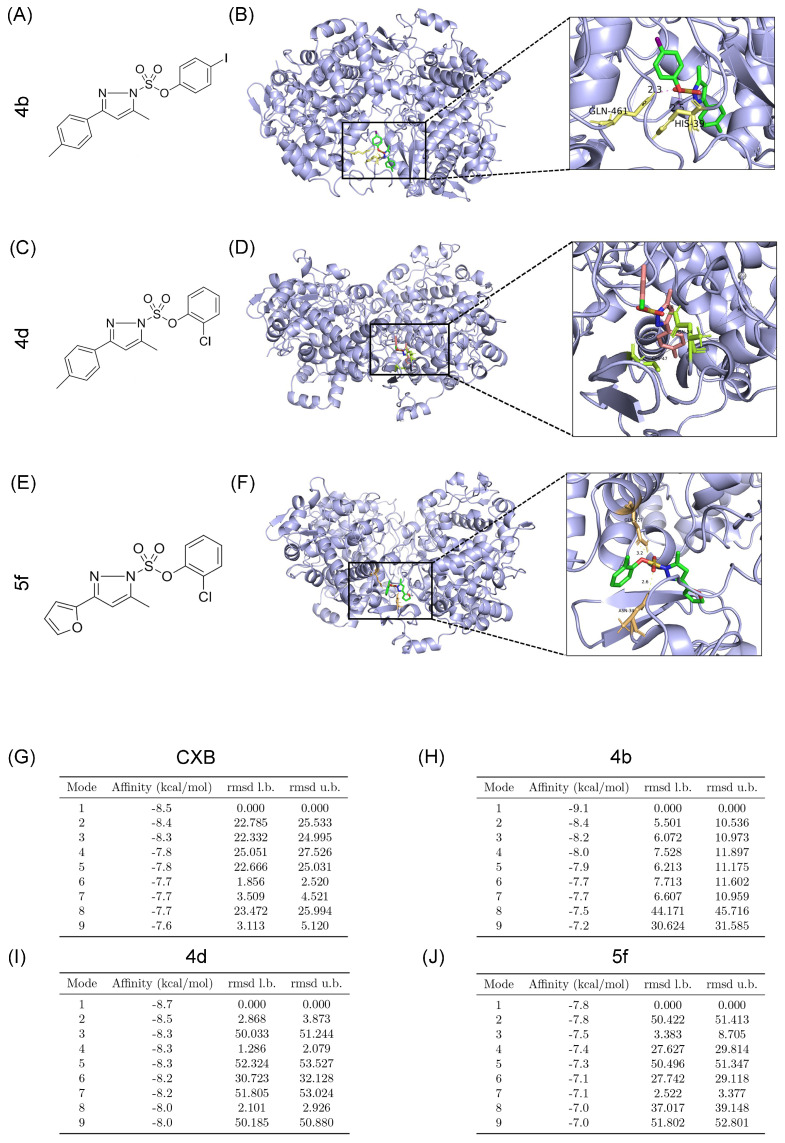
Molecular docking of compounds **4b**, **4d**, and **5f** with COX-2. (**A**) Structure of **4b**. (**B**) Three-dimensional structure of **4b**–COX-2 molecular docking. Yellow and pink dotted lines represent hydrogen bonds. Hydrogen atoms are not shown for clarity. (**C**) Structure of **4d**. (**D**) Three-dimensional structure of **4d**–COX-2 molecular docking. Yellow and pink dotted lines represent hydrogen bonds. Hydrogen atoms are not shown for clarity. (**E**) Structure of **5f**. (**F**) Three-dimensional structure of **5f**–COX-2 molecular docking. Yellow and pink dotted lines represent hydrogen bonds. Hydrogen atoms are not shown for clarity. (**G**) Alignment of lowest binding energy for molecular docking of CXB–COX-2. (**H**) Alignment of lowest binding energy for molecular docking of **4b**–COX-2. (**I**) Alignment of lowest binding energy for molecular docking of **4d**–COX-2. (**J**) Alignment of lowest binding energy for molecular docking of **5f**–COX-2.

Molecular docking simulations demonstrated that compounds **4b** (Figure 1A), **4d** (Figure 1C), and **5f** (Figure 1E) effectively bind to the active site of COX-2 [38], yielding optimal 3D docking conformations (Figure 1B,D,F). The lowest binding energies for compounds **4b**, **4d**, and **5f** were −9.1, −8.7, and −7.8 kcal/mol, respectively (Figure 1H–J), compared to −8.5 kcal/mol for CXB (Figure 1G).

### 2.2. Cytotoxicity Evaluation of Compounds **4b**, **4d**, and **5f** on OSCC Cell Lines

#### 2.2.1. CCK-8 Assay and ${\mathrm{IC}}_{50}$ Determination of Compounds **4b**, **4d**, and **5f** in OSCC Cells

**Figure 2:**
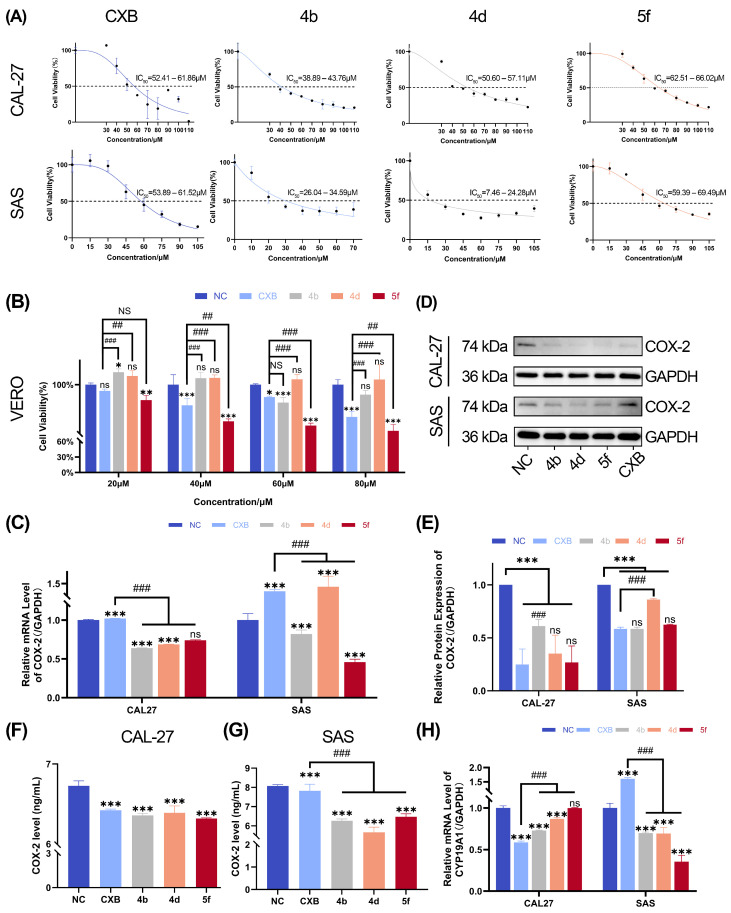
Cytotoxicity and COX-2 inhibition by CXB and derivatives in OSCC cell lines. (**A**) CCK-8 assay showing proliferation inhibition and ${\mathrm{IC}}_{50}$ values in CAL-27 and SAS cells treated with CXB, **4b**, **4d**, and **5f** for 24 h. (**B**) CCK-8 assay assessing cytotoxicity of working concentrations of CXB, **4b**, **4d**, and **5f** in VERO cells. (**C**) RT-qPCR analysis of COX-2 mRNA levels in CAL-27 (left) and SAS (right) cells after 24 h-treatment. Expression of COX-2 protein in CAL-27 and SAS cells was determined by Western blot. (**D**) Representative Western blot images. (**E**) Quantitative analysis of the results. COX-2 levels in CAL-27 (**F**) and SAS cells (**G**) were measured with ELISA. (**H**) RT-qPCR analysis of CYP19A1 mRNA levels in CAL-27 (left) and SAS (right) cells post-treatment. Data are shown as mean and SD of triplicates (mean ± SD). (* *p* < 0.05, ** *p* < 0.01, *** *p* < 0.001; ns: no significance vs. NC. ^##^ *p* < 0.01, ^###^ *p* < 0.001; NS: no significance vs. CXB).

Our first step was carefully establishing the optimal concentration able to impact 50% of the OSCC cell viability, and both CXB and compounds **4b**, **4d**, and **5f** inhibited proliferation rates on CAL-27 cells, where the ${\mathrm{IC}}_{50}$ values were 52.41–61.86 μM, 38.89–43.76 μM, 44.27–55.09 μM, and 52.85–74.83 μM for CXB, **4b**, **4d**, and **5f**, respectively. Notably, compounds **4b**, **4d**, and **5f** displayed a marked dose-dependent inhibition across the tested concentration ranges (Figure 2A). Consistent with the previous findings, the SAS cells exhibited comparable dose-dependent responses, demonstrating the ${\mathrm{IC}}_{50}$ values were 53.89–61.52 μM, 26.04–34.59 μM, 7.46–24.28 μM, and 59.39–69.49 μM for CXB, **4b**, **4d**, and **5f**, respectively (Figure 2A). Standardized working concentrations were then established based on ${\mathrm{IC}}_{50}$ values. The final working concentrations on CAL-27 cells were 60 μM, 30 μM, 60 μM, and 60 μM for CXB, **4b**, **4d**, and **5f**, respectively, and 80 μM, 40 μM, 80 μM, and 80 μM on SAS cells. These concentrations were used in all subsequent mechanistic assays (RT-qPCR, Western blot, ELISA, migration, apoptosis, and JAK/STAT pathway analyses) unless otherwise specified.

#### 2.2.2. CCK-8 Assay for Inhibitory Effects of Compounds **4b**, **4d**, and **5f** on VERO Cells

The CCK-8 assay was employed to evaluate the relative cell viability of VERO cells (African green monkey kidney cells) treated with varying concentrations of compounds **4b**, **4d**, and **5f** for 24 h. The results demonstrated that at the working concentrations, the proliferation rates of VERO cells in the compound **4b**- and **4d**-treated groups remained largely unchanged compared to the control group, whereas compound 5f exhibited a moderate inhibitory effect on cell proliferation (Figure 2B). To further assess the safety profile and selectivity of the compounds, the selectivity index (SI) was calculated as the ratio of IC_50_ in normal cells (VERO) to IC_50_ in cancer cells (CAL-27 or SAS): SI = IC_50,VERO_/IC_50,cancer_. Values greater than 1 indicate preferential activity against cancer cells over normal cells [39,40,41,42]. In CAL-27 cells, the SI values for CXB, **4b**, **4d**, and **5f** were 4.27, 2.47, >1.86, and 3.50, respectively; in SAS cells, they were 4.22, 3.40, >6.00, and 3.50 (Table 1).

### 2.3. Validation of Inhibitory Effects of CXB and Compounds **4b**, **4d**, and **5f** on the Key Gene COX-2

Based on the molecular design rationale of the compounds, we wanted to verify the expression status of COX-2 in OSCC cells after being treated with each compound. RT-qPCR was performed to investigate the inhibitory effects after a 24 h treatment. The results revealed a significant downregulation of COX-2 mRNA levels in all compound-treated groups. Furthermore, the mRNA expression levels in the **4b**-, **4d**-, and **5f**-treated groups were significantly lower than those in the CXB-treated group, confirming the superior inhibitory efficacy of these compounds on COX-2 gene expression (Figure 2C). In SAS cells, treatment with compounds **4b** and **5f** significantly downregulated the mRNA expression levels of the COX-2 gene compared to the control group. Conversely, treatment with compound **4d** and CXB induced a marked upregulation of COX-2 mRNA expression (Figure 2C).

Western blotting analysis demonstrated that in CAL-27 cells, treatment with compounds **4b**, **4d**, and **5f** significantly reduced COX-2 protein expression compared to both the control group and the CXB-treated group (Figure 2D,E). In SAS cells, all compound-treated groups exhibited a marked decrease in COX-2 protein levels relative to the control group. Notably, CXB treatment resulted in an even greater suppression of COX-2 expression in SAS cells compared to the other compounds (Figure 2D,E).

ELISA results demonstrated that in CAL-27 cells, the COX-2 protein levels in treatment groups were slightly reduced compared to the control group (Figure 2F). In SAS cells, no marked difference in COX-2 expression was observed between the control and CXB-treated groups (Figure 2G). However, a substantial reduction in COX-2 levels was detected in the compound-treated groups relative to both the control and CXB-treated groups (Figure 2G).

These findings further validated the robust inhibitory effects of the tested compounds on COX-2 expression.

### 2.4. Inhibition of OSCC Cell Proliferation by Compounds ***4b***, ***4d***, and ***5f***

As reported above, the CCK-8 assay demonstrated that all compounds exerted inhibitory effects on both CAL-27 and SAS cells at the working concentrations (Figure 2A). To explore the molecular mechanisms underlying the anti-proliferative effects of these compounds on OSCC cell lines, RT-qPCR was performed to quantify the mRNA expression levels of proliferation-related genes.

In CAL-27 cells, compared to the control group, the mRNA expression of the CYP19A1 gene was significantly downregulated in the **4b**- and **4d**-treated groups, whereas compound **5f** slightly influenced the expression, although not significantly. On the other hand, all compound-treated groups exhibited markedly lower expression levels than the CXB-treated group (Figure 2H). Moving on to consider SAS cells, the mRNA expression levels of CYP19A1 in cells treated with compounds **4b**, **4d**, and **5f** were significantly reduced compared to both the control group and the CXB-treated group, further confirming the consistent suppression of CYP19A1 by these compounds across distinct OSCC cell models (Figure 2H).

### 2.5. Inhibition of OSCC Cell Migration by Compounds **4b**, **4d**, and **5f**

**Figure 3:**
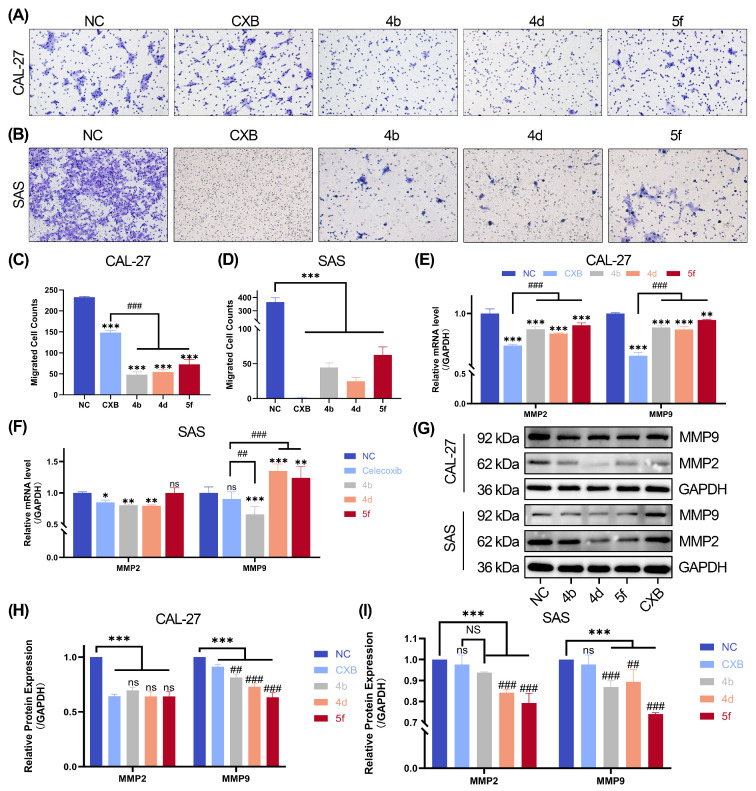
Compounds **4b**, **4d**, and **5f** inhibit OSCC cell migration. (**A**) Transwell migration assay in CAL-27 cells treated with compounds **4b**, **4d**, and **5f**, and the quantitative analysis of the results. (**B**) Transwell migration assay in SAS cells treated with compounds **4b**, **4d**, and **5f**, and the quantitative analysis of the results. (**C**) The quantitative analysis of the Transwell migration assay results in CAL-27 cells. (**D**) The quantitative analysis of the Transwell migration assay results in SAS cells. (**E**,**F**) mRNA expression levels of MMP2 and MMP9 in CAL-27 (**E**) and SAS (**F**) cells by RT-qPCR. The protein expressions were assessed by Western blot in CAL-27 cells. Representative Western blot images (**G**) and quantitative analysis of the results (**H**). The protein expression was assessed by Western blot in SAS cells. Representative Western blot images (**G**) and quantitative analysis of the results (**I**). Data are shown as mean and SD of triplicates (mean ± SD). (* *p* < 0.05, ** *p* < 0.01, *** *p* < 0.001; ns: no significance vs. NC. ^##^ *p* < 0.01, ^###^ *p* < 0.001; NS: no significance vs. CXB).

Transwell migration assays showed that treatment with compounds **4b**, **4d**, and **5f** significantly reduced the migratory capacity of CAL-27 (Figure 3A) and SAS (Figure 3B) cells, as evidenced by a decreased number of cells crossing the basement membrane compared to the control group (Figure 3C,D). To investigate the molecular mechanisms underlying these anti-migratory effects, RT-qPCR and Western blotting assays were performed. RT-qPCR revealed that 24 h treatment with compounds **4b**, **4d**, and **5f** significantly downregulated the mRNA expression of COX-2, MMP2, and MMP9 in CAL-27 cells compared to the control group. Strikingly, the CXB-treated group exhibited even lower MMP2 and MMP9 mRNA levels than the compound-treated groups (Figure 3E). Western blotting analysis further corroborated these findings: expression of MMP2 was reduced relative to the control in all groups and differed significantly from CXB, whereas MMP9 expression was decreased relative to both the control and CXB groups (Figure 3G,H).

In SAS cells, compounds **4b**, **4d**, and CXB markedly suppressed MMP2 mRNA expression compared to the control group. In contrast, the **5f**-treated group showed no statistically significant suppression of the mRNA expression of MMP2 (Figure 3F). Moreover, for the MMP9 gene, only the 4b-treated group displayed a significant reduction in mRNA levels (Figure 3F). Interestingly, Western blot analysis indicated that all compound-treated groups exhibited decreased MMP2 and MMP9 protein expression compared to the control group, despite the absence of consistent suppression of the MMP9 gene (Figure 3G,I).

### 2.6. Compounds **4b**, **4d**, and **5f** Promote Apoptosis in OSCC Cell Lines

**Figure 4:**
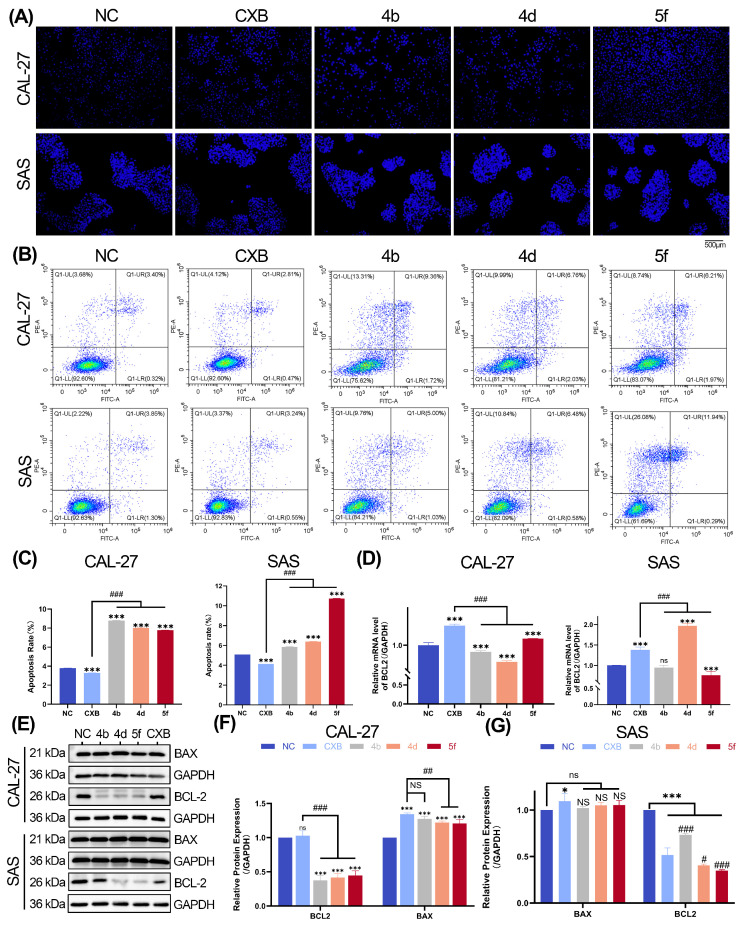
Compounds **4b**, **4d**, and **5f** promote apoptosis in OSCC cells. (**A**) Hoechst 33342 staining of CAL-27 and SAS cells following treatment with CXB and the compounds. Apoptotic cells are characterized by condensed nuclei with intense staining. Schematic diagram of cell apoptosis measured with flow cytometry analysis. After treatment with CXB and compounds for 24 h, the apoptosis of CAL-27 and SAS cells was detected via Annexin V-FITC/PI staining. (**B**) Representative flow cytometry scatter plots. (**C**) The statistical results of the apoptotic rate. (**D**) Relative mRNA expression levels of BCL2 in CAL-27 and SAS cells by RT-qPCR. (**E**,**F**) Apoptosis-related protein expression assessed by Western blot. CAL-27 cells: Representative Western blot images (**E**) and quantitative analysis of the results (**F**). (**E**,**G**) SAS cells: Representative Western blot images (**E**) and quantitative analysis of the results (**G**). Data are shown as mean and SD of triplicates (mean ± SD). (* *p* < 0.05, *** *p* < 0.001; ns: no significance vs. NC. ^#^ *p* < 0.05, ^##^ *p* < 0.01, ^###^ *p* < 0.001; NS: no significance vs. CXB).

Hoechst 33342 staining was performed to assess apoptosis in CAL-27 and SAS cells treated with compounds **4b**, **4d**, and **5f** (treatment groups), CXB, or the control group. Fluorescence microscopy revealed a significant increase in apoptotic cells in the CXB-, **4b**-, **4d**-, and **5f**-treated groups compared to the NC groups (Figure 4A).

Quantitative analysis using the Annexin V-FITC Apoptosis Detection Kit demonstrated that the total apoptotic cell population (early and late apoptosis) was significantly higher in the **4b**-, **4d**-, and **5f**-treated groups compared to the control group, with CXB exhibiting comparable pro-apoptotic efficacy (Figure 4B,C).

To investigate the molecular mechanisms underlying compound-induced apoptosis, transcriptional and translational profiling was conducted. In CAL-27 cells, RT-qPCR analysis demonstrated that 24 h treatment with compounds **4b** and **4d** significantly downregulated BCL2 mRNA levels compared to the control group, surpassing the inhibitory effect of CXB. In contrast, compound **5f** upregulated BCL2 transcription relative to the NC group yet remained lower than CXB (Figure 4D). Western blotting analysis corroborated these findings, revealing decreased protein levels of the anti-apoptotic factors BCL2, alongside increased expression of the pro-apoptotic protein BAX in all compound-treated groups. Notably, compounds **4b**, **4d**, and **5f** exhibited stronger suppression of BCL2 than CXB (Figure 4E,F).

In SAS cells, compounds **4b** and **5f** significantly suppressed BCL2 mRNA expression compared to both the NC and CXB groups, whereas compound 4d paradoxically increased BCL2 transcription (Figure 4D). Despite this transcriptional divergence, Western blotting analysis consistently demonstrated reduced BCL2 protein levels across all compound-treated SAS cells (Figure 4E,G). Although BAX expression rose slightly in all treated groups, the changes were not statistically significant relative to the NC group (Figure 4E,G).

### 2.7. Effects of Compounds **4b**, **4d**, and **5f** on the JAK/STAT Signaling Pathway

**Figure 5:**
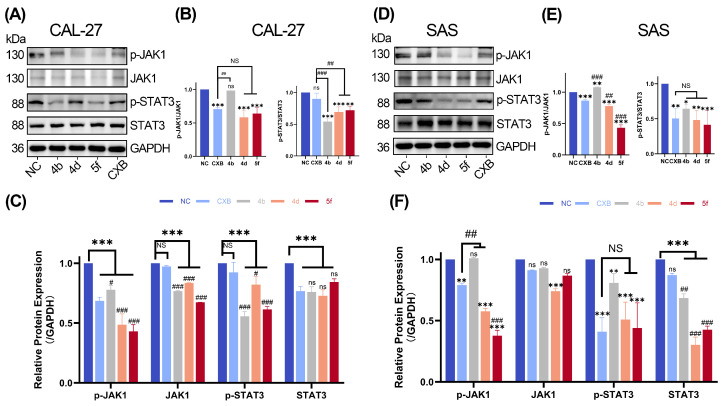
Effects of Compounds **4b**, **4d**, and **5f** on JAK/STAT Signaling Pathway. (**A**–**C**) The protein expression was assessed by Western blot in CAL-27 cells. Representative Western blot images (**A**) and quantitative analysis of the results (**B**,**C**). (**D**–**F**) The protein expression was assessed by Western blot in SAS cells. Representative Western blot images (**D**) and quantitative analysis of the results (**E**,**F**). Data are shown as mean and SD of triplicates (mean ± SD). (* *p* < 0.05, ** *p* < 0.01, *** *p* < 0.001; ns: no significance vs. NC. ^#^ *p* < 0.05, ^##^ *p* < 0.01, ^###^ *p* < 0.001; NS: no significance vs. CXB).

Western blotting demonstrated a marked reduction in protein levels of JAK1, phosphorylated JAK1 (p-JAK1), STAT3, and phosphorylated STAT3 (p-STAT3) in CAL-27 cells following compound treatment (Figure 5A–C). In SAS cells, p-JAK1 levels were reduced by all compounds except **4b**, which showed no significant change. Total JAK1 trended downward in every treatment group, but only **4d** reached statistical significance versus NC. p-STAT3 was uniformly decreased across all treatments, and total STAT3 was likewise reduced, with a greater extent of suppression than CXB (Figure 5D–F). These alterations in both transcriptional and post-translational regulators of the JAK/STAT pathway were statistically significant across experimental groups, suggesting potent inhibition of pathway activation by the tested compounds.

## 3. Discussion

As one of the most prevalent malignancies in the head and neck region, OSCC is primarily managed through multi-modal therapies combining surgery with chemoradiotherapy [43,44,45]. However, conventional treatments face limitations, including systemic toxicity, suboptimal efficacy, and drug resistance [46]. As an inducible inflammatory mediator activated within the tumor microenvironment, COX-2 drives cancer progression by promoting angiogenesis and suppressing apoptosis [47,48]. Accumulating evidence identifies COX-2 as a critical therapeutic target in OSCC. Shariq et al. demonstrated that COX-2 overexpression correlates significantly with poor prognosis in OSCC patients, suggesting that targeted inhibition of COX-2 may improve clinical outcomes [49]. Aparnadevi et al. further investigated COX-2 expression in OSCC via immunohistochemistry, revealing a progressive increase in COX-2 staining intensity with advancing tumor stage [50]. Preclinical studies underscore the therapeutic potential of COX-2 inhibition in OSCC. Yoshihiro et al. reported that administration of a COX-2 inhibitor in SAS-LM3 tumor-bearing mice (an OSCC xenograft model) suppressed lymphangiogenesis and lymphatic metastasis [51]. Similarly, Qian et al. demonstrated that combined therapy with cetuximab (an epidermal growth factor receptor [EGFR] monoclonal antibody) and the COX-2 inhibitor CXB significantly reduced tumor volume in OSCC xenograft models [28]. Corporately, these findings establish COX-2 as a pivotal regulator of OSCC pathogenesis, progression, and metastasis, providing a robust scientific rationale for precision therapies targeting this pathway.

Previous studies have established that NSAIDs and selective COX-2 inhibitors exhibit therapeutic efficacy against various cancers [52,53,54]. For instance, Elena et al. demonstrated that the use of NSAIDs and Coxibs significantly reduces the incidence risk of colorectal cancer and colon adenomas by approximately 50% [55]. Similarly, Agrawal et al. reported a 20% reduction in breast cancer risk associated with NSAID administration [56]. Building on these findings, our research group previously analyzed structural modification strategies for a series of nonsteroidal compounds, including selective COX-2 inhibitors, leading to the design of pyrazole N-aryl sulfonate derivatives [57,58]. In vitro experiments confirmed that compounds **4b** and **4d** exhibit excellent anti-inflammatory and analgesic activities, coupled with low oral toxicity. These properties establish a strong foundation for their development as lead compounds for managing inflammation and pain [38]. However, their molecular mechanisms of action remain incompletely elucidated, and their efficacy in other tumor models has not been validated.

In this study, compounds **4b**, **4d**, and **5f** were systematically evaluated for their effects on OSCC cell lines, with the aim of unraveling their mechanisms in modulating OSCC cell behavior. We used a molecular docking approach to verify compounds could bind to COX-2 protein to form a stable structure and the conjugates were stable. Moreover, integrated analysis of RT-qPCR, Western blot, and ELISA results validated the potent inhibitory effects of the tested compounds on COX-2 gene expression in both CAL-27 and SAS cells. These multi-modal findings collectively demonstrate the robust suppression of COX-2 signaling in OSCC cell lines. The anti-proliferative effects of compounds **4b**, **4d**, and **5f** on OSCC cell lines were assessed using the CCK-8 assay. Results demonstrated a significant dose-dependent inhibition of cell proliferation across the tested concentration range. RT-qPCR analysis revealed that these compounds markedly downregulated mRNA expression of the proliferation-associated gene CYP19A1 in CAL-27 cells, suggesting that suppression of CYP19A1 transcription may underlie their anti-proliferative activity.

Furthermore, in VERO cells, compounds **4b**, **4d**, and **5f** showed lower cytotoxicity than CXB at working concentrations, indicating a more favorable safety profile. Selectivity indices were highest for CXB in CAL-27 and for 4d in SAS. Notably, all SI values exceeded 1.0, indicating preferential activity against OSCC cells relative to normal cells. Notably, use of VERO cells for preliminary selectivity estimation constitutes a limitation due to limited tissue relevance, and future validation in human normal oral squamous epithelial cells is planned. These findings support the therapeutic potential of the tested compounds in OSCC treatment, combining targeted efficacy with reduced off-target toxicity.

The matrix metalloproteinase (MMP) family comprises a class of zinc-dependent endopeptidases characterized by a conserved ${\mathrm{Zn}}^{2+}$-containing catalytic domain [59,60]. These enzymes degrade extracellular matrix (ECM) components and play pivotal roles in regulating cellular proliferation, migration, differentiation, and signaling pathways [61,62]. Previous studies have identified COX-2 as a key mediator of tumor metastasis, primarily through its stimulation of inflammatory mediators that upregulate MMP2 and MMP9 expression, thereby enhancing the metastatic potential of cancer cells [59,63,64]. Our findings corroborate the functional interplay between COX-2, MMP2, and MMP9 in OSCC pathogenesis. Transwell migration assays demonstrated that compounds **4b**, **4d**, and **5f** significantly inhibited the migratory capacity of CAL-27 and SAS cells. Molecular profiling revealed that these compounds downregulated the mRNA expression of MMP2 in both CAL-27 and SAS cells, suggesting suppression of MMP2 transcription as a primary mechanism for anti-migratory effects. Moreover, both in CAL-27 and SAS cells, we further observed a concurrent reduction in MMP9 mRNA and protein levels following compound treatment, indicating dual transcriptional and translational inhibition of MMP9. These data jointly suggest that the anti-metastatic activity of compounds **4b**, **4d**, and **5f** is mediated through a COX-2-dependent regulatory axis. By inhibiting COX-2 expression, these derivatives suppress downstream MMP2 and MMP9 activation, thereby attenuating the invasive potential of OSCC cells.

Programmed apoptosis, a critical process for maintaining tissue homeostasis, is governed by the BCL2 protein family, which comprises both pro-apoptotic and anti-apoptotic members that collectively determine cellular fate [65,66]. In this study, Hoechst 33342 staining revealed a significant increase in apoptotic cells across **4b**-, **4d**-, and **5f**-treated OSCC groups compared to controls, as visualized by fluorescence microscopy. Flow cytometry further quantified apoptosis in CAL-27 and SAS cells following 24 h compound treatment. The total apoptotic cell population was significantly elevated in all compound-treated groups, with **4b** exhibiting the strongest pro-apoptotic effect in CAL-27 cells, while **5f** demonstrated optimal efficacy in SAS cells. These cell line-specific disparities suggest distinct molecular mechanisms of action, warranting further mechanistic exploration and structure-activity optimization for enhanced tumor selectivity. To delineate the apoptotic mechanisms, RT-qPCR and Western blotting analyses were performed. In CAL-27 cells, compounds **4b** and **4d** suppressed BCL2 mRNA expression and BCL2 protein levels, concurrently upregulating pro-apoptotic BAX expression. Similarly, in SAS cells, **4b**, **4d**, and **5f** downregulated BCL2 transcription and translation while synergistically enhancing BAX expression. These findings indicate that the compounds induce OSCC apoptosis via coordinated suppression of the COX-2/BCL2 axis and activation of BAX, with BCL2 emerging as a central molecular target.

Emerging evidence positions COX-2 as a pivotal regulator within multifaced oncogenic signaling cascades [67]. COX-2 has been shown to modulate tumor cell proliferation, migration, metabolism, and angiogenesis through control of various genes associated with key pathways, including JAK/STAT3, WNT/β-catenin/TCF, and PI3K/AKT [68,69,70]. Among these, the JAK/STAT3 axis plays a critical role in cellular processes such as growth, survival, differentiation, and pathogen resistance [71]. STAT3, a transcription factor constitutively activated in numerous human malignancies, undergoes phosphorylation by Janus kinases (JAKs), facilitating its homodimerization, nuclear translocation, and subsequent activation of oncogenic transcripts, including anti-apoptotic factors like BCL2 [72,73]. In this study, Western blot analyses of JAK/STAT3 signaling components revealed that compound treatment significantly downregulated JAK1 and STAT3 expression in CAL-27 and SAS cells. Concurrently, protein levels of p-JAK1, JAK1, p-STAT3, and STAT3 were markedly reduced in CAL-27 cells. Similar suppressions of relevant proteins were observed in SAS cells. These findings suggest that the compounds attenuate JAK1/STAT3 phosphorylation, thereby inhibiting downstream transcriptional activation of BCL2 and impairing OSCC cell proliferation, invasion, and metastasis. Our data further reinforce the hypothesis that COX-2 serves as a critical upstream modulator of the JAK/STAT3 pathway. However, the precise molecular interplay between COX-2 and JAK/STAT3 remains unresolved and warrants targeted investigation using other approaches.

Despite these encouraging results, several limitations warrant consideration. First, only two OSCC cell lines (CAL-27 and SAS) were examined, which does not fully reflect the molecular and phenotypic heterogeneity of OSCC; future work will incorporate additional models, including patient-derived primary cultures and organoids, to improve representativeness. Second, all functional assays were performed in vitro and therefore do not recapitulate the complexity of the tumor microenvironment—particularly stromal and immune interactions that can influence drug response. Further more, in vivo evaluation such as orthotopic or genetically engineered murine models is still required to determine pharmacokinetics, efficacy, and potential off-target effects under physiological conditions. Finally, the primary objective of subsequent work will be to delineate the precise mechanisms of these compounds in OSCC by systematically perturbing the JAK/STAT3 axis through targeted genetic and pharmacological inhibition strategies.

## 4. Materials and Methods

### 4.1. Molecular Docking

Using the PDB database (Protein Data Bank, https://www.rcsb.org, accessed on 7 May 2025 ), retrieve the 3D structure of COX-2 protein [74]. Based on the known chemical formulas of COX-2 inhibitors **4b**, **4d**, and **5f**, generate corresponding 3D structures using the Swiss Target Prediction database. Import the processed receptor and ligand structures into Autodock Vina (Version 1.5.7) for hydrogen addition and docking calculations. Use −5 kcal/mol as the inclusion/exclusion criterion: a binding energy less than −5 kcal/mol indicates good binding. Visualize the molecular docking results using PyMOL (Version 3.1.4.1).

### 4.2. Preparation of Working Solutions

An appropriate volume of 2 mg/mL compound emulsion was dissolved in 0.1% DMSO (Dimethyl sulfoxide) to prepare a 200 μg/mL stock solution [38]. Prior to experimental use, the working concentration of the compound was adjusted with culture medium. The prepared working solution was subjected to ultrasonication for 30 min (ultrasonic disperser parameters: frequency 40 kHz, power 240 W; SB-5200DTD, SCIENTZ, Ningbo, China) to ensure homogeneous dispersion before experimental procedures.

### 4.3. Cell Culture

The OSCC cell lines CAL-27 (STCC12901P, Servicebio, Wuhan, China) and SAS (CC0706, Cellcook, Guangzhou, China) were cultured in Dulbecco Modified Eagle Medium F12 (DMEM/F12) (G4610-500ML, Servicebio, Wuhan, China) supplemented with 10% fetal bovine serum (FBS) (AB-FBS-DIA1050, ABW, Shanghai, China) and 100 U/mL penicillin-streptomycin-gentamicin solution (G4014-100ML, Servicebio, Wuhan, China). VERO cells were cultured in DMEM (G4524-500ML, Servicebio, Wuhan, China) supplemented with 10% FBS (AB-FBS-DIA1050, ABW, China) and 100 U/mL penicillin–streptomycin–gentamicin solution (G4014-100ML, Servicebio, Wuhan, China). Celmoderatels were maintained at 37 °C in a humidified 5% CO_2_ incubator. For experiments, 1 × 10^6^ cells were seeded per T-25 culture flask.

### 4.4. RT-qPCR

Total RNA was extracted from treated cells at the predefined working concentrations (CAL-27: CXB 60 μM, **4b** 30 μM, **4d** 60 μM, **5f** 60 μM; SAS: CXB 80 μM, **4b** 40 μM, **4d** 80 μM, **5f** 80 μM) using the SPARKeasy Cell RNA Kit (AC0202-A, Sparkjade, Jinan, China). Equal amounts of RNA were reverse transcribed into cDNA using the Reverse Transcription Kit (AG11705, Accurate Biology, Guangzhou, China) following the manufacturer’s instructions. Quantitative PCR was performed on a QIAGEN Rotor-GeneQ system (9001862, QIAGEN, Hilden, Germany) with SYBR Green Pro Taq HS Premix II (AG11702, Accurate Biology, China).

The PCR protocol consisted of initial denaturation at 95 °C for 30 s, followed by 40 cycles of 95 °C for 5 s and 60 °C for 30 s. All reactions were performed in triplicate, with melting curve analysis confirming amplification specificity. Primer sequences are provided in Table 2. The expression of the target genes was normalized to GAPDH, and fold changes were calculated in the manner of the $2^{-\Delta \Delta \mathrm{CT}}$.

### 4.5. Cell Proliferation Assay

CAL-27, SAS, and VERO cells were seeded in 96-well plates at a density of 5000 cells per well. At specified time intervals, 10 μL of Cell Counting Kit-8 (CCK-8) reagent (K1018, Apexbio, Boston, MA, USA) was added to each well and incubated for 2 h. Absorbance at 450 nm was measured using a microplate reader (Infinite M200 Pro, Tecan Group, Männedorf, Switzerland).

### 4.6. Cellular Migration Assay

Following 24 h drug treatment at the predefined working concentrations, CAL-27 and SAS cells (5 × $10^{5}$ cells/mL in serum-free medium) were seeded in Transwell upper chambers (200 μL/well, 1 × $10^{5}$ cells) with 700 μL complete medium containing 10% FBS in lower chambers. After 36 h of incubation (37 °C, 5% CO_2_), the cells from the upper surface of the membrane were wiped off using a cotton swab, whereupon migrated cells were fixed (4% paraformaldehyde [P0099-100 mL, Beyotime, Haimen, China], 4 °C, 4 h), stained (0.1% crystal violet [C0121-100 mL, Beyotime, China], 30 min), and quantified by counting five random fields under a microscope (CX23, Olympus, Tokyo, Japan) using ImageJ (v1.8.0.322), with triplicate wells per condition.

### 4.7. Hoechst 33342 Staining

After 24 h drug treatment at the predefined working concentrations, CAL-27 and SAS cells were dyed with 300 μL Hoechst 33342 staining (C1022, Beyotime, China) in the dark at 37 °C for 10 min. Then, the residual Hoechst 33342 staining was washed 3 times with 1 × phosphate-buffered saline (PBS, G4250-500ML, Servicebio, China). The images were then immediately examined under an inverted fluorescence microscope (CX23, Olympus, Japan).

### 4.8. Flow Cytometry (FCM) Analysis—Measurement of Apoptosis

After 24 h drug treatment at the predefined working concentrations, CAL-27 and SAS cells were trypsinized, centrifuged (200× *g*, 5 min, RT), and washed twice with 1 mL ice-cold PBS. Cell aliquots ((5–10) × $10^{4}$ cells) were resuspended in 195 μL Annexin V-FITC binding buffer, followed by sequential addition of 5 μL Annexin V-FITC and 10 μL propidium iodide (PI) (BMS500FI-100, Thermo Fisher Scientific, Wien, Austria) with gentle mixing. After 10–20 min of incubation in darkness (20–25 °C) with intermittent resuspension (2–3 times), samples were placed on ice and analyzed within 1 h using a flow cytometer (CytExpert software [Version 2.4.0.28, Beckman Coulter, Brea, CA, USA]). Triplicate independent experiments were performed for statistical analysis.

### 4.9. Enzyme-Linked Immunosorbent Assay (ELISA)

With the aim of determining COX-2 concentrations in OSCC cell lines after treatment at the predefined working concentrations, we used a specialized ELISA kit (F0564-A, FANKEW, Shanghai, China), following the manufacturer’s instructions. Briefly, 40 μL of the sample dilution and 10 μL of the sample (sample final dilution is 5-fold) were added to testing sample wells and incubated for 30 min at 37 °C. After that, discard the liquid and wash the plate using washing buffer. Repeat 5 times, drying by patting. Subsequently, samples were incubated with 50 μL of HRP-conjugate reagent in each well for 30 min at 37 °C. Wash 5 times, then add Chromogen Solution A and B and incubate for 10 min at 37 °C. Lastly, 50 μL of stop solution was added to each well to stop the reaction, and the sample absorbance was measured using a microplate reader at 450 nm after adding stop solution within 15 min.

### 4.10. Western Blotting Analysis

Cell lysates were centrifuged at 12,000× *g* for 10 min at 4 °C and then quantified using the BCA Protein Assay Kit (CW0014S, Cwbio, Taizhou, China). The lysate was denatured with sodium dodecyl sulfate–polyacrylamide gel electrophoresis (SDS-PAGE) sample loading buffer (P1040, Solarbio, Beijing, China), followed by SDS-PAGE and electrotransfer to polyvinylidene difluoride membranes (10600023, Cytiva, Marlborough, MA, USA). The membranes were incubated overnight at 4 °C with GAPDH (1:5000, 1049-1-AP, Proteintech Group, Wuhan, China), anti-PTGS2 (1:2000, 12375-1-AP, Proteintech Group, China), anti-BAX (1:1000, ab777, Abcam, Cambridge, UK), anti-BCL2 (1:5000, ET1702-53, HUABIO, Hangzhou, China), anti-BCL-XL (1:8000, 10783-1-AP, Proteintech Group, China), anti-MMP2 (1:3000, 66366-1-Ig, Proteintech Group, China), anti-MMP9 (1:3000, 10372-2-AP, Proteintech Group, China), anti-JAK1 (1:3000, 66466-1-Ig, Proteintech Group, China), anti-phospho-JAK1 (1:400, GB115604-100, Servicebio, China), anti-STAT3 (1:2000, 10253-2-AP, Proteintech Group, China), and anti-phospho-STAT3 (1:1000, 39595, Activemotif, Shanghai, China) at the appropriate dilution and then incubated with the secondary antibody at room temperature for 1.5 h. The bands were visualized by employing an ECL detection reagent (36208ES76, Yeasen, Shanghai, China) and independently quantified twice using ImageJ (v1.8.0.322) and ChemiScope Analysis (Version 2.1.6.0) software. Subsequently, the obtained data were normalized to GAPDH.

### 4.11. Statistical Analysis

Descriptive statistics were presented as mean ± standard deviation. Statistical differences between groups were assessed using two-way ANOVA (Analysis of Variance). A *p*-value < 0.05 was considered to be notably different. All analyses were performed using GraphPad Prism software (version 10.1.2, San Diego, CA, USA).

## 5. Conclusions

In summary, our study demonstrates that the novel pyrazole N-aryl sulfonate derivatives—potent selective COX-2 inhibitors—effectively suppress proliferation, migration, and survival in CAL-27 and SAS cells, likely through inhibition of the JAK/STAT3 signaling axis. These compounds represent promising lead candidates for OSCC-targeted therapy, offering a dual mechanism of action that combines COX-2 suppression with downstream blockade of pro-survival pathways. By bridging the gap between COX-2 biology and JAK/STAT3-driven oncogenesis, this work provides a strategic framework for developing precision therapies against therapy-resistant OSCC subtypes.

## Figures and Tables

**Table 1:** IC_50_ values and SI of CXB and compounds **4b**, **4d**, and **5f**.

Compounds	IC_50_/μM			Selectivity Index	
	CAL-27	SAS	VERO	CAL-27	SAS
**CXB**	57.01	57.64	243.3	4.27	4.22
**4b**	41.42	30.16	102.4	2.47	3.40
**4d**	53.88	16.68	>100	>1.86	>6.00
**5f**	64.25	64.20	225.0	3.50	3.50

A favorable SI > 1.0 indicates a drug with efficacy against tumor cells greater than the toxicity against normal cells.

**Table 2:** The primer sequences of the target genes.

Genes	Forward Primer (5′-3′)	Reverse Primer (5′-3′)
*VEGFα*	CATCCAATCGAGACCCTGGTG	TTGGTGAGGTTTGATCCCCATA
*CYP19A1*	CCTTGTTCGTATGGTCACAGTCT	CGTGTTAGAGGTGTCCAGCAT
*MMP2*	CTCATCGCAGATGCCTCGAA	TTCAGGTAATAGGCACCCTTGAAGA
*MMP9*	ACCTTCACTCGCGTGTACAG	GGACCACAACTCGTCATCGT
*STAT3*	GCAGCTGACTACACTGGCAGAGA	ATTGTCCAGCCAGACCCAGAA
*JAK2*	TTGAAGACCGGGATCCTACACA	AGGGTCATACCGGCACATCTC
*BCL2*	CACTGAGATTTCCACGCCGAAG	TTTCTCGGCACAATTGGTAGCTT
*PTGS2*	TCCCTTGGGTGTCAAAGGTAAA	TGGCCCTCGCTTATGATCTG
*GAPDH*	GGAAGCTTGTCATCAATGGAAATC	TGATGACCCTTTTGGCTCCC

## Data Availability

The original contributions presented in this study are included in the article. Further inquiries can be directed to the corresponding authors.

## Abbreviations

The following abbreviations are used in this manuscript:

AKT	Protein kinase B
ANOVA	Analysis of variance
BAX	BCL2-associated X protein
BCL2	B-cell lymphoma 2
BCL-XL	BCL2-like protein 1
CAFs	Cancer-associated fibroblasts
CCK-8	Cell Counting Kit-8
COX-2	Cyclooxygenase-2
CSC	Cancer stem cell
CXB	Celecoxib
DMEM	Dulbecco Modified Eagle Medium
ELISA	Enzyme-linked immunosorbent assay
FBS	Fetal bovine serum
GAPDH	Glyceraldehyde-3-phosphate dehydrogenase
HRP	Horseradish peroxidase
JAK	Janus kinase
JAK1	Janus kinase 1
MMP2	Matrix metalloproteinase 2
MMP9	Matrix metalloproteinase 9
NSAIDs	Non-steroidal anti-inflammatory drugs
OSCC	Oral squamous cell carcinoma
PBS	Phosphate-buffered saline
PGE2	Prostaglandin E2
PI	Propidium iodide
PI3K	Phosphoinositide 3-kinase
PTGS2	Prostaglandin-endoperoxide synthase 2

p-JAK1	Phosphorylated Janus kinase 1
p-STAT3	Phosphorylated STAT3
RT-qPCR	Real-time quantitative polymerase chain reaction
SDS-PAGE	Sodium dodecyl sulfate-polyacrylamide gel electrophoresis
SD	Standard deviation
SI	Selectivity Index
STAT3	Signal transducer and activator of transcription 3
TCF	T-cell factor
TME	Tumor microenvironment
WNTβ	WNT/β-catenin signaling pathway
